# The International Medical Congress at Rome

**Published:** 1894-04-07

**Authors:** 


### The International Medical Congress at Rome.

(By our own Correspondent.)

Rome was never more full of doctors than it is at the present time. The list of visitors for Easter has been an exceptionally heavy one, and that, taken with the Congress, will give some idea of the number of persons here.

The representative for England and the United States is Dr. Rochet, who speaks English to perfection, and renders to our countrymen the very greatest assistance. He is well-known also for his ecclesiastical work, and is both priest and physician. Many English practitioners are here, including Sir Spencer Wells, Sir William MacCormac, Sir Dyce Duckworth, Messrs. Makins, Howard, Marsh, Morgan, F. Semon, J. Althaus, G. Cornell, Priestly, H. Radcliffe-Crocker, Ferrier, A. George Harley, G. G. Bantock, Lauder Brunton, and Henry Power. Four languages, English, French, German, and Italian are those in which all communications of the Congress will take place.

The building, barely ready for the inauguration of the 28th, is very handsome. One half of it is given up to the Exhibition and the other to the Academy of Fine Arts.

The buildings in which the details of the Congress take place are merely improvised wooden sheds and buildings. The members of the Congress receive as a mark of distinction a blue rosette, and a marguerite (in graceful allusion to the Queen of Italy) for the ladies. Presidents and members of the Foreign National Committees wear distinctive badges also.

The bronze medal given to each member of the Congress is the production of Stephen Johnson, of Milan.

### The Opening Ceremony.

The opening of the Medical Congress took place on Thursday, the 29th ult., at ten a.m. in the Costanzi Theatre, in the presence of their Majesties the King and Queen of Italy. The theatre was full to suffocation. The Queen looked well. She was dressed in a straw-coloured costume, with circular cape of the same trimmed with light green satin, a pink bonnet with a white aigrette. Unfortunately no band enlivened the proceedings. On either side of their Majesties were ranged the representatives of foreign powers, ambassadors, &c. Signor Crispi welcomed the members of the Congress in the name of the Italian Government, and the Syndic, Prince Emanuele Raspoli, in the name of the citizens. The Hon. Bacelli, Minister of Public Instruction, then delivered the opening discourse in Latin.

The special medical representatives from foreign nations made each an address. Virchow, of Berlin, on presenting himself was received with tremendous cheers. Sir Wm. MacCormac spoke for Great Britain. The representative from Spain, dressed in a black gown with amber-coloured hood and amber-coloured hat of the shape of a gigantic bell with a handle to it, was remarkably conspicuous. Some twenty representatives each delivering an address, which was in the greater number of instances imperfectly heard, protracted the proceedings to an inordinate length, and half of the building was empty before the departure of the King and Queen. The former remained standing during the whole proceedings. At the termination of the official opening the several special medical representatives were presented to their Majesties.

The afternoon of Thursday was occupied at the Polyclinique in the constitution of Presidents and Secretaries of the several sections. It is undoubtedly a great drawback having the Administration building, viz., the Polyclinique, so far away. It is outside the walls of Rome, at the back of the Military Camp, and reached by a long drive passing out through the Porta Pia.

In the Section of Anatomy the name of Dr. Cunningham appears as one of the Vice-Presidents; and in the Section of Dermatology the names of Malcolm Morris, Crocker, Pringle, and Thin.

The exhibits are small in number and very poor. Germany is most to the fore with antiseptic appliances, England is poorly represented with an exhibit of concentrated medicines in capsules, and at the British Medical Association meetings a better show is made. The gallery of the building was reserved for literary publications, and from here a communication existed to the New Gallery of Fine Arts containing a collection of modern paintings. The first reception took place at half past nine p.m. The effect was much spoilt by the non-insistence of evening dress, the English visitors only being without exception in full evening dress. The German and Italian officers of the army and navy gave the only brightness to the assembly. One of the regimental bands played during the evening. The crowd was very large.

Dr. Rochet, who was easily recognizable by wearing the arms of Great Britain as a badge on his coat, is most indefatigable in his exertions for the comfort of the English visitors. The English navy is represented by Dr. MacDonald, and the army by Dr. Notter.

Among the most illustrious Germans we note Professor Rudolph Virchow, of Berlin. A larger number of persons have declared their intention of being present at this Congress than has been present at any previous one. They are divided as follows: Italian—Members of Congress, 2,560; ladies, 158; invited, 14. Foreign—Members of Congress, 3,269; ladies, 810; invited, 52.

Several banquets and a gala performance at the theatre are announced, and are open to members of the Congress on payment.

The mismanagement of the Congress is most marked. The confusion at the several sections of the Polyclinic is dreadful, and culminated yesterday by the English surgeons sending in a protest. The weight of the discontent falls largely upon the shoulders of Mr. Makins, Secretary for England, but it is not his fault, for he is probably the ablest organizer, and the most courteous gentleman in the profession, but that of the Italian Executive, which has displayed little business or organizing power. Everybody's sympathy will be extended to Mr. Makins, in the trying circumstances, remembering his splendid services at the International Congress in London, the conspicuous success of which was largely due to his devotion and skill as an administrator.

The numbers who have attended are three times as many as were expected, and the arrangements have broken down under the strain. Moscow has been decided upon as the seat of the next International Congress, if, as one of the chief movers said to me, there is ever another.

The gala representation at the Costanzi Theatre consisted in a performance of "La Traviata," chosen for the doctors, as an history of *une malade*. The vocal and instrumental part was good, but the play was monotonous, and the performance too long.

The Clinico Medico, situated near the Castle of St. Angelo, was visited by the Congress members. It contained some fifty patients. The wards consisted of two large and long rooms, very clean and healthy. The patients up and about were clothed in white garments, and everything carried out on the latest most approved hygiene manner, the tops of the bedside tables being made of glass. Listerism has done much in instilling cleanliness into foreign nations as regards the sick and injured.

A banquet was given on the 29th by the Obstetrical and Gynæcology Society, at the Hotel Quirinal to the representatives from other nations.

To show how things are managed, several packets of the invitation tickets for the lunch at the baths of Caracalla, have been stolen, and it has been found necessary to cancel the whole of them, and to issue fresh tickets.

One member who has been to the Polyclinic on two occasions to read his paper, was twice put off. The distance from the city is very great, and cabs difficult to get, and expensive. The bright spot in management is the *Lancet* office, where one receives every kindness from their representative, and whose rooms might very well be called the English Reception and Committee Room.

### List of Papers to be Read by Englishmen at the Congress.

Lauder Brunton—On the Physiological Action of Various Compounds of Nitrogen in Relation to their Chemical Constitution.

Burney Yeo—The Management of Fevers, and Particularly of Typhoid or Enteric Fever.

Andrew Clark—Is Pneumonia an Inflammation?

Dr. C. Black—On the Treatment of Acute Nephritis and the Pathological Significance of Albuminuria in Relation to Life Insurance.

Kendal Franks—Loreta's Operation for the Cure of Stricture of the Œsophagus.

Mayo Robson (of Leeds)—Surgical Treatment of Spina Bifida and Meningocele, with Reports of Cases where Excision has been Performed; Surgical Removal of Supra-renal Tumour; Surgery of Bile Ducts.

E. Owen—The Treatment of Paralytic Talipes Equino-Varus in Children.

W. Stokes—Bone Tunnelling in Tuberculous Arthritis of the Hip Joint.

C. Ball (of Dublin)—Radical Cure of Hernia by Torsion of Sac, Improved Method.

G. Buchanan (of Glasgow)—Radical Cure of Hernia.

G. H. Makins—An Operation for the Relief of Epispadias in the Female.

Dr. R. Barnes—The Physiology and General Pathology Illustrating the Study of Gestation.

Dr. Clement Godson—How will the Introduction of the Operation of Symphsiotomy into Practice affect Cæsarian Section and its Modifications.

Dr. G. G. Bantock—Supra-vaginal Hystercetomy: The Treatment of the Pedicle.

Thomas Bryant.—Prolapse of the Urethra in the Female.

H. Power—A Case of Gunshot Wound of the Orbit with Subsequent Aneurism Requiring Ligature of the Carotid Artery. Recovery with loss of vision in one eye.

George Cowell—Is Iridectomy in the Extraction of Hard Cataract Advantageous?

J. D. Macdonald—As to What Provision can be Made for the Better Management of the Sick and Wounded During Action in Modern Ships.

S. Osborn—The Extension of the Ambulance Movement throughout Italy, by means of the Italian Branch of the Order of St. John.

E. Hart—On the Sanitation of the Indian Outports, and of Mecca, as a Means of Preventing an Eruption of Cholera into Europe; Cholera Nurseries and their Suppression.

W. R. Smith—The Ambulance Service for Infectious Diseases in London; Effects of Filtration upon the Presence of Micro-organisms in Drinking Water.

A. H. Allbutt—Infant Mortality and Premature Death.

C. R. Drysdale—High Birth-rates Considered as the Principal Cause of Premature Death in European states.

Fortescue Fox—The Sulphur Waters of Great Britain.

Abraham Phineas—A New Method of Treating Parasitic Diseases of the Scalp; Some Drawings of Rare Conditions of the Skin.

Dr. D. Savill—On a New Form of Epidemic Skin Disease, with photographs, illustrations, microscopic, and other specimens.

Malcolm Morris—The Lichen Question.

Johnston Lavis—The Gulf of Naples as a Winter Resort for English and Americans.

